# Supplementary material for: Dynalign II: common secondary structure prediction for RNA homologs with domain insertions
Source: Nucleic Acids Res. 2014 Nov 21;42(22):13939–48. doi: 10.1093/nar/gku1172 (PMC4267632; doi:10.1093/nar/gku1172)
Supplement: SUPPLEMENTARY DATA [file supp_gku1172_nar-02021-z-2014-File012.zip › manual/Text/DuplexFold.html]

RNAstructure Command Line Help: DuplexFold


|  |  |  |
| --- | --- | --- |
|  | RNAstructure Command Line Help DuplexFold | - Contents - Index |
| DuplexFold is used to predict lowest free energy structures containing two strands. The structures will not contain intramolecular pairs. Note that output is written to a CT file where the sequences are concatenated, with an intermolecular linker between them ("III"). USAGE: DuplexFold <seq file 1> <seq file 2> <ct file> [options] Required parameters: <seq file 1> \| The name of a sequence file containing input data for the first sequence. Note that lowercase nucleotides are forced single-stranded in structure prediction.; <seq file 2> \| The name of a sequence file containing input data for the second sequence. Note that lowercase nucleotides are forced single-stranded in structure prediction.; <ct file> \| The name of a CT file to which output will be written. Options which don't require added values: -d, -D, --DNA \| Specify that the sequence is DNA, and DNA parameters are to be used. Note that the backbone type must be the same for both sequences. Default is to use RNA parameters.; -h, -H, --help \| Display the usage details message. Options which require added values: -l, -L, --loop \| Specify the maximum number of unpaired nucleotides in an internal or bulge loop. Default is 6 unpaired nucleotides.; -m, -M, --maximum \| Specify a maximum number of structures. Note that suboptimal structures are generated until either the maximum number of structures are reached or the maximum percent difference is reached (below). Default is 10 structures.; -p, -P, --percent \| Specify a maximum percent difference in folding free energy change in suboptimal structures. Note that suboptimal structures are generated until either the maximum percent free energy difference is reached or until the maximum number of structures is reached (above). Default is 40 percent (specified as 40, not 0.4).; -t, -T, --temperature \| Specify the temperature at which calculation takes place in Kelvin. Default is 310.15 K, which is 37 degrees C.; -w, -W, --window \| Specify a window size for generating suboptimal structures. Smaller windows (down to zero) will result in a larger set of structures that are similar. Larger windows result in fewer structures that are more different in predicted pairs. Default is 0 nucleotides. References 1. Reuter, J.S. and Mathews, D.H. "RNAstructure: software for RNA secondary structure prediction and analysis." *BMC Bioinformatics*, 11:129. (2010). 2. Piekna-Przybylska, D., DiChiacchio, L., Mathews, D.H. and Bambara, R.A. "A sequence similar to tRNA3Lys gene is embedded in HIV-1 U3/R and promotes minus strand transfer." *Nature Structural & Molecular Biology*, 17:83-89. (2010). | | |
| Visit The Mathews Lab RNAstructure Page for updates and latest information. | | |
